# Longitudinal white matter hyperintensity changes and cognitive decline in patients with minor stroke

**DOI:** 10.1007/s40520-021-02024-5

**Published:** 2022-01-27

**Authors:** Jingwen Jiang, Kanmin Yao, Xiaojun Huang, Yu Zhang, Fanxia Shen, Suiqing Weng

**Affiliations:** 1 Department of Neurology and Institute of Neurology, Rui Jin Hospital, Shanghai Jiao Tong University School of Medicine, Shanghai, China; 2 Department of Radiology, Rui Jin Hospital, Shanghai Jiao Tong University School of Medicine, Shanghai, China; 3 Department of Neurology, Shanghai Minhang Hospital, Shanghai Fu Dan University, Shanghai, China

**Keywords:** White matter hyperintensities, Minor stroke, Dynamic change, Cognitive impairment

## Abstract

**Background and objective:**

As reported, both minor stroke and white matter hyperintensities (WMHs) are associated with an increased risk of cognitive impairment and dementia. The underlying factors for dynamic changes in WMH volume and cognitive performances in patients with minor stroke remain poorly understood. A 2-year longitudinal study was designed to investigate the factors associated with the changes in white matter hyperintensity (WMH) volume on brain MRI and cognitive decline in patients with minor stroke.

**Methods:**

A group of eligible patients with minor ischemic stroke was recruited in a row. At the initial and 2-year follow-up visits, all the participants underwent routine examinations, multimodal MRI, and cognitive assessment. Using a lesion prediction algorithm tool, we were able to automate the measurement of the change in WMH volume. During the 2-year follow-up, cognitive function was evaluated using Telephone Interview for Cognitive Status-Modified (TICS-m). Participants’ demographic, clinical, and therapeutic data were collected and statistically analyzed. Regression analyses were used to test the relationships between risk factors and changes in WMH volume and cognitive decline.

**Results:**

Finally, we followed up with 225/261 participants for 2 years, with a mean age of 65.67 ± 10.73 years (65.6% men). WMH volume was observed to be increased in 113 patients, decreased in 74 patients, and remained stable in 58 patients. Patients with WMH progression were more often had a history of hypertension (*p* = 0.006) and a higher CSVD burden both at baseline and follow-up visit (*p* < 0.05). Longitudinally, the proportion of patients taking antihypertension medications on a regular basis in the regression group was higher than in the stable group (*p* = 0.01). When compared to the stable group, the presence of lacunes (OR 9.931, 95% CI 1.597–61.77, *p* = 0.014) was a stronger predictor of progression in WMH volume. 87 subjects (38.6%) displayed incident cognitive impairment. The progression of WMH volume was a significant risk factor for cognitive decline (*p* < 0.001).

**Conclusions:**

The longitudinal change of WMH is dynamic. The regressive WMH volume was associated with the use of antihypertensive medications on a regular basis. The presence of lacunes at the initial visit of the study was a stronger predictor of WMH progression. The progression of WMH volume could be useful in predicting cognitive decline in patients with minor stroke.

## Introduction

Minor stroke is proposed to define as National Institutes of Health Stroke Scale ≤ 3 with normal level of consciousness and favorable short- and medium-term outcomes [1]. Minor ischemic strokes account for approximately 30% of new strokes in China [2]. Growing epidemiological studies show that patients with transient ischemic attack (TIA) or minor stroke could experience residual impairments [3], reduced quality of life [4], and cognitive impairment [5]. White matter hyperintensities (WMHs) of presumed vascular origin are a common feature on brain MRI in patients with manifested arterial disease [6] as well as normal elderly individuals [7]. WMHs are highly prevalent (28.4–78.5%) in the elderly and Asian people according to a population-based survey [8]. As reported, WMHs have been linked to an increased risk of developing cognitive impairment and dementia [9, 10].

Previous population-based longitudinal studies found that WMH volume could increase or decrease [11, 12]. Thus, the changes in WMH volume over time were suggested to be dynamic, and the etiology of WMH volume progression or shrinkage is not fully explained. As imaging techniques advance, several automated and semi-automated methods based on various algorithms could be chosen to segment and quantify WMH volumes. The effect of WMH changes on cognitive decline in minor stroke patients is poorly understood [13]. Therefore, the goal of our study was to investigate the factors underlying changes in WMH volume as measured by automated segmentation methods at baseline and 2 years later. Second, we aimed to examine the impact of CSVD (cerebral small vessel disease)-related imaging parameters on cognitive decline in patients who had a minor stroke.

## Methods

### Study design and participants

Patients with minor ischemic stroke who presented to the Neurology Outpatient Department or admitted to the Neurology ward of Ruijin Hospital between January 2016 and December 2018 were prospectively and consecutively recruited in our study. We defined minor ischemic stroke as a focal onset of neurological symptoms lasting > 24 h, with a National Institutes of Health Stroke Scale (NIHSS) score of ≤ 3 and not expected to result in dependency (modified Rankin Scale (mRS) score < 3). The diagnosis of stroke was confirmed by an expert panel based on clinical findings and MRI. The Shanghai Jiao Tong University School of Medicine Affiliated Ruijin Hospital’s Institutional Review Board approved the study. Participants who could provide consent were included from general practices and we excluded patients with severe systemic diseases limiting movement prior to stroke, white matter lesions caused by immune or hereditary causes, communication difficulties due to dysphasia or dysarthria, and hemorrhagic stroke. All the subjects underwent routine examinations, vascular risk factor assessment, brain MRI scan, and neuropsychological tests at the time of initial assessment (baseline) and 2 years after the index stroke.

### MRI acquisition

Brain MRI scans were performed on a 3.0 T scanner (Philips 3.0 T Achieva; Philips Health care) using the following sequence obtained: axial T1-weighted, axial T2-weighted, fluid-attenuated inversion recovery (FLAIR), diffusion-weighted imaging, and susceptibility-weighted imaging. All of the above sequences of the initial MRI scan should be the same as those of the follow-up scan in a patient. To maintain scanner uniformity, daily quality assurance tests were performed by three radiologists with kappa value of 0.82. The following parameters were applied to the 2D FLAIR images: axial slice thickness of 2 mm; TR/TE/TI = 4800/125/1650 ms, and a voxel size of 0.96 × 0.95 × 3.00mm^3^. The 3D T1-weight parameters included TR/TE = 7.9/4.5 ms and a voxel size of 1.0 × 1.0 × 1.0 mm^3^.

### Measurement of longitudinal WMH volume

For the present study, FLAIR images were registered to T1-weighted images by applying SPM12 (Welcome Institute of Neurology, University College London, UK, http://www.fil.ion.ucl.ac.uk/spm/doc/) for Matlab (The MathWorks, Inc., Natick, Massachusetts, United States). White matter hyperintensities (WMHs) were focal or confluent hyperintensities in the deep and periventricular white matter on FLAIR images, and we used the modified Fazekas scale to visually rate the degree of WMH severity [14]. Automated WMH segmentation was performed on the registered 2D FLAIR images by the lesion prediction algorithm ([15], Chapter 6.1) as implemented in the LST toolbox version 3.0.0 (www.statistical-modelling.de/lst.html) for SPM. The quantification of WMH volume was performed using the spatial dimensions of the voxels in each MRI slice.

The longitudinal WMH volume change between the baseline and the follow-up FLAIR images was measured. WMH volume changes were interpreted as (1) progression, (2) stable, and (3) regression. Ultimately, we defined the progression or regression of WMH change group as more than 0.25 ml increase or decrease observed. The stable group represented less than ± Δ of 0.25 ml of WMH lesion volume. The threshold was derived from the data of Cho described in 2015 to discriminate groups [16].

### Measurement of cerebral small vessel disease burden

Two trained vascular neurologists independently appraised all of the available scans for the presence and severity of CSVD characteristics. Lacunes were defined as round-shaped cerebrospinal fluid isointense lesions of ≤ 20 mm in diameter surrounded by a zone of parenchyma with increased signal intensity on T2-weighted and FLAIR images [17]. Enlarged perivascular spaces (EPVS) were featured as ≤ 2 mm round or linear isointense lesions, while manifesting as hyperintense lesions on T2-weighted images and hypointense signals on T1/FLAIR sequences [18]. Susceptibility-weighted imaging classified cerebral microbleeds as homogeneous rounded lesions with signal loss within a diameter < 10 mm [19]. The higher burden of CSVD was defined as presence of two or more of the following imaging characteristics: ≥ 1 asymptomatic lacune; periventricular WMH Fazekas score 3 or if deep WMH Fazekas score 2 or 3; moderate-to-extensive (10–25 or > 25) EPVS in the basal ganglia; ≥ 1 deep CMBs [20]. The inter-observer agreement (Kappa value) was 0.882 for higher burden of CSVD.

### Cognitive function assessment

All participants’ cognitive function was assessed using the modified Telephone Interview for Cognitive Status (TICS-m) via face-to-face or telephone interview at both the baseline and 2-year follow-up visits. TICS-m is a telephone adaption of the Mini-Mental State Examination that assesses overall cognitive performance, with scores ranging from 0 (worst performance) to 40 (best performance) representing memory, orientation, and verbal and attention function, respectively [21]. Participants who missed one or more follow-up phone calls were considered lost to follow-up. The subjects were classified as having no cognitive impairment (21 < TICS-m sore ≤ 40), mild cognitive impairment (13 < TICS-m score ≤ 20), or moderate cognitive impairment with a TICS-m score of 12 or higher [22]. Thus, incident cognitive decline was defined as a transition from no cognitive impairment to mild cognitive impairment in patients with no cognitive impairment, and cognitive status deterioration from mild to moderate extent in patients with mild cognitive impairment.

### Statistical analysis

Data are presented as *n*(%) for categorical variables or as mean ± SD for parametric data after employing the Kolmogorov–Smirnov test for normality test. The Chi-square test or Fisher’s exact test were used to compare categorical variables, while the Student’s *t* test was used to compare continuous variables. The total scores of TICS-m from baseline to the end of the follow-up period were used to assess cognitive decline. We used a forward stepwise logistic regression analysis with *p* < 0.05 (as shown in Table 1) to evaluate the effect of the variables at baseline on longitudinal change of the WMH volume after adjusting for age and sex. We also analyzed the association between the MRI CSVD indicators, medical medications, and cognitive deterioration using logistic regression analyses. The statistically significant difference level was set at *p* < 0.05. All statistical analyses were conducted using SPSS version 24 (IBM Corp., Chicago, IL).

**Table 1:** Demographic, clinical, and imaging characteristics of participants determined by white matter hyperintensities volumes

	WMH progression (*N* = 113)	WMH stable (*N* = 58)	WMH regression (*N* = 74)	*p* value
Age at visit	66.84 ± 10.03	68.61 ± 11.60	66.91 ± 10.61	0.291
Male, *n*(%)	76(67.3)	35(60.3)	48(64.9)	0.883
Education (years)	8.49 ± 2.27	9.94 ± 2.41	8.82 ± 1.87	0.054
Vascular risk factors				
Hypertension, *n*(%)	99(87.6)	38(65.5)	46(62.2)	***0.006***
Diabetes mellitus, *n*(%)	52(46.0)	13(22.4)	28(37.8)	0.159
Coronary artery disease, *n*(%)	18(15.9)	10(17.2)	4(5.4)	0.306
Atrial fibrillation, *n*(%)	5(4.4)	0(0)	0(0)	0.335
Current smoking, *n*(%)	17(15.0)	7(12.1)	18(24.3)	0.433
Drinking, *n*(%)	15(13.3)	6(10.3)	11(14.9)	0.937
Hyperlipidemia, *n*(%)	20(17.7)	7(12.1)	2(2.7)	0.098
Large vessel occlusion	54(47.8)	19(32.8)	26(35.1)	0.328
Regular medications				
Antiplatelets, *n*(%)	54(47.8)	32(55.2)	22(29.7)	0.112
Statins, *n*(%)	37(32.7)	16(27.6)	24(32.4)	0.916
Antihypertension agents, *n*(%)	39(34.5)	7(12.1)	33(44.6)	***0.02***
Antidiabetic agents, *n*(%)	20(17.8)	5(8.6)	13(17.6)	0.475
Baseline SVD markers				
WMH volume	12.95 ± 8.52	3.59 ± 7.07	12.53 ± 7.94	***0.027***
Extensive WMHs, *n*(%)	51(45.1)	29(50.0)	48(64.9)	0.170
Presence of lacunes, *n*(%)	83(73.5)	16(27.6)	43(58.1)	***0.000***
Presence of CMBs, *n*(%)	36(31.9)	19(32.8)	13(17.6)	0.286
Moderate- and high-grade BG-EPVS, *n*(%)	26(23.0)	10(17.2)	15(20.3)	0.818
Higher burden of CSVD, *n*(%)	76(67.3)	16(27.6)	48(64.9)	***0.008***
Follow-up SVD markers				
WMH volume	22.14 ± 9.52	3.61 ± 7.08	9.64 ± 8.36	***0.000***
Extensive WMHs, *n*(%)	60(53.1)	39(67.2)	50(67.6)	0.297
Presence of lacunes	96(85.0)	23(39.7)	48(64.9)	***0.000***
Presence of CMBs	42(37.2)	19(32.8)	17(23.0)	0.385
Moderate- and high-grade BG-EPVS	39(34.5)	10(17.2)	15(20.3)	0.175
Higher burden of CSVD	86(76.1)	16(27.8)	43(58.1)	***0.000***

*WMH* white matter hyperintensities; *extensive WMHs* periventricular WMH (Fazekas 3) or deep WMH (Fazekas 2 + 3); *BG-EPVS* basal ganglia enlarged perivascular spaces; *CMBs* cerebral microbleeds; CSVD: cerebral small vessel disease

Higher Burden of CSVD: the presence of two or more of the following imaging characteristics: ≥ 1 asymptomatic lacune; periventricular WMH Fazekas score 3 or if deep WMH Fazekas score of 2 or 3

Bold and italic values significant with *p* < 0.05

## Results

### Sociodemographic and clinical characteristics

261 patients with clinically confirmed minor ischemic stroke were recruited at the outset. Six patients were excluded due to deterioration to mRS > 3 at the 2-year mark. Lost to follow-up within the 2 years were 30 patients. Finally, the 225 participants who completed our study’s full follow-up were 65.67 ± 10.73 years old (65.6% men) and had 8.79 ± 2.23 years of education. The demographics and clinical characteristics are summarized in Table 1. At baseline, 183 (81.3%) of the patients had hypertension, 93 (41.3%) had diabetes, 29 (12.9%) had hyperlipidemia, 32 (14.2%) patients had coronary artery disease, and 5 participants had atrial fibrillation. 42 (18.6%) were current smokers and 32 (14.2%) consumed alcohol regularly.

### CSVD markers on MRI

The prevalence [*n*(%)] of CSVD neuroimaging markers in this cohort at initial scanning was as follows: *n* = 142(63.1%) had lacunes, *n* = 68(30.2%) had more than one CMBs, and *n* = 51(22.7%) had moderate-to-extensive EPVS in the basal ganglia. During the follow-up period, 113 patients showed a progression of WMH and 74 patients had WMH volume regression. There was no discernible change in WMH volume observed in 58 patients. Except for taking antihypertension agents, there were no significant differences in frequencies between the progression, stable, and regression groups in sex, education, or medications. Patient with WMH progression had a higher frequency of hypertension history (*p* = 0.006). In terms of SVD markers, the progression group had a larger WMH volume, along with higher frequencies of the presence of lacunes and a higher burden at both the baseline and follow-up visits (*p* < 0.05) (Table 1).

### Factors associated with longitudinal WMH change

Longitudinally, the WMH volume decreased in people who regularly took antihypertension medications compared to the stable group (OR 3.359, 95% CI 2.016–5.599, *p* = 0.01). The presence of lacunes (OR 9.931, 95% CI 1.597–61.77, *p* = 0.014) conferred a stronger predictor of progression in WMH volume compared to the stable group (Table 2).

**Table 2:** Multinomial logistic regression results for changes in WMH volume

Independent variables	Progression vs. stable				Regression vs. stable			
	Odds ratio	O.R. (95% CI)		*p*	Odds ratio	O.R. (95% CI)		*p*
History of hypertension	1.873	0.463	7.569	0.379	0.401	0.088	1.818	0.236
Antihypertension agents	0.449	0.133	1.521	0.199	3.359	2.016	5.599	***0.010***
Baseline SVD makers								
WMH volume	1.03	0.935	1.133	0.551	1.025	0.928	1.133	0.624
Presence of lacunes	9.931	1.597	61.77	***0.014***	4.933	0.986	24.681	0.052
Higher burden of CSVD	0.982	0.187	5.175	0.983	0.899	0.138	5.847	0.911

Bold and italic values significant with *p* < 0.05 by adjusting for age and sex

### The presence of CSVD markers and incident cognitive decline

Finally, 86.2% (225/261) of the population completed the 2-year follow-up. At year 2, 87 subjects (38.6%) displayed incident cognitive impairment, as shown in Table 3. We found no differences in age, gender, years of education, or initial cognitive status between the cognitive decline and no cognitive decline groups. Subjects who eventually having cognitive decline were more likely to have a higher burden of CSVD, as well as the presence of lacunes and CMBs at the initial visit (*p* < 0.05). Furthermore, cognitive decliners had a higher frequency of WMH volume progression (*p* < 0.05) (Table 3).

**Table 3:** Demographic and imaging characteristics of participants determined by cognitive status

	Incident cognitive decline (*N* = 87)	No cognitive decline (*N* = 158)	*p*
Age at visit	68.18 ± 9.66	64.57 ± 11.04	0.244
Male, *n* (%)	64 (73.6)	98 (62.0)	0.209
Education (years)	8.50 ± 2.27	8.92 ± 2.21	0.961
Baseline CSVD markers			
Higher burden of CSVD, *n* (%)	69 (79.3)	71 (45.0)	***0.006***
Extensive WMHs, *n* (%)	44 (50.6)	84 (53.2)	0.859
Presence of lacunes, *n* (%)	60 (69.0)	82 (51.9)	***0.031***
Presence of CMBs, *n* (%)	37 (42.5)	31 (19.6)	***0.006***
Moderate- and high-grade BG-EPVS, *n* (%)	25 (28.7)	26 (16.5)	0.073
Baseline median TICS-m score (IQR)	34 (30.00–34.00)	35.00 (33.00–35.00)	0.82
Regular medications			
Antiplatelets, *n* (%)	33 (37.9)	75 (47.5)	0.193
Statins, *n* (%)	27 (31.0)	50 (31.6)	0.560
Antihypertension agents, *n* (%)	28 (32.2)	51 (32.3)	0.832
Antidiabetic agents, *n* (%)	16 (18.4)	22 (13.9)	0.590
Changes in WMH volume, *n* (%)			
Progression	63 (72.4)	50 (31.6)	***0.007***
No progression (stable/regression)	41 (47.1)	91 (57.6)	

Bold and italic values significant with *p* < 0.05

Then, we examined the impact of baseline CSVD markers and progression in WMH volume on cognitive decline over time (as shown in Table 4). The subjects with WMH progression had significantly worse cognitive status in model 1 adjusted for socio-demographic factors (age, sex, and education years), as well as in models further adjusted for vascular risk factors (*p* < 0.001) (as shown in Table 4).

**Table 4:** The effects of CSVD markers on incident cognitive decline

CSVD-related variables	Model 1		Model 2	
	Estimate (95% CI)	*p* value	Estimate (95% CI)	*p* value
Presence of lacunes	− 0.10 (− 0.21, 0.01)	0.07	− 0.10 (− 0.21, 0.01)	0.07
Presence of CMBs	− 0.08 (− 0.19, 0.03)	0.14	− 0.08 (− 0.19, 0.03)	0.14
Higher burden of CSVD	− 0.05 (− 0.16, 0.06)	0.35	− 0.05 (− 0.16, 0.06)	0.35
Progressive WMH volume	− 0.11 (− 0.15, − 0.07)	** < *0.001***	− 0.11 (− 0.15, − 0.07)	** < *0.001***

Bold and italic values significant with *p* < 0.05

Model 1: Adjusted for age, gender, and education and initial cognitive status

Model 2: Adjusted for age, gender, education and initial cognitive status, current smoking, alcohol intake, hypertension, diabetes, hyperlipidemia, history of heart disease, and large vessel occlusion

## Discussion

Over a 2-year period, dynamic changes in WMH integrity, CSVD imaging markers, and cognitive function were primarily observed in patients with a history of minor stroke in our study. The main findings of the study were as follows: (1) among the 225 participants who completed follow-up, 113 patients (50.2%) progressed, 74 patients (32.8%) regressed, and 58 patients (25.8%) remained stable in WMH volume over a 2-year period; (2) WMH regression was associated with regular use of antihypertension medications; presence of lacunes at baseline was a strong predictor of WMH progression; (3) 87 subjects (38.6%) displayed incident cognitive impairment; and (4) subjects with WMH volume progression performed significantly worse on the cognitive impairment.

The longitudinal change in WMH volume has mostly been reported as progression [23, 24], whereas several studies have reported a decrease in participants with ischemic stroke over time [12, 16]. Other studies have found that the dynamic change in WMH remained stable [25, 26]. In our study, no significant differences in demographics or baseline vascular risk factors were observed in the progression and regression groups compared to the stable group,which is consistent with the previous studies [13, 27]. The decrease in WMH volume could be due to measurement errors or alternative and mixed hypotheses of leukoaraiosis, such as perivascular demyelination and spongiosis [28]. In our study, significant differences in the regular use of antihypertension medications were observed between the regression and stable groups. The longitudinal observation studies which were conducted in community-dwelling elderly individuals revealed arterial hypertension was a strong predictor of WMH volume progression and antihypertensive treatment was related to a smaller increase in WMH volume [29]. As a result, adequate antihypertention treatment may slow the progression of WMH. Our study concluded that the presence of lacunes at baseline was associated with WMH volume progression. Previous longitudinal studies found that WMH and lacunes progressed over time in minor stroke patients and tissues adjacent to WMH might be susceptible to further ischemia including formation of lacunes [15].

Aside from the negative effect on cognitive function, previous research has linked WMH to increased infarct growth, stroke severity, stroke recurrence, and poor functional outcome in patients suffering from moderate to severe stroke [30, 31]. In community-dwelling elderly individuals, WMH progression has been reported to be associated with cognitive decline [32–34]. There has been little research into the impact of dynamic WMH change on cognitive function in minor stroke population. Higher WMH volume has been demonstrated to be associated with longer symptom duration in patients with minor stroke [35]. Furthermore, the effect of age on cognitive function in the elderly or stroke survivors is largely debatable. A prospective cohort study named Oxford Vascular Study (OXVASC) discovered that high WMH load in patients over the age of 80 with a previous TIA or minor stroke was strongly associated with cognitive impairment.

The strength of our study was that patients with minor stroke were recruited prospectively, and neuroimaging parameters as well as cognitive function were measured repeatedly at a 2-year follow-up. We quantify the WMH volume using an automated WMH segmentation methods, LST-LPA, which has been shown to perform well both within and across scanners in a multicenter dataset just followed KNN-TTP [36]. However, the TICS-m is not as sensitive as detailed neuropsychological tests and may miss mild cognitive decline. The findings of our study should be confirmed in larger sample size studies, as well as studies with a longer follow-up period.

## Conclusions

Our findings support the notion that the change in WMH volume was dynamic, as patients with a history of minor stroke could show progression, regression, or stability. WMH regression was linked to regular use of antihypertension medications, and the presence of lacunes at baseline was a strong predictor of WMH progression. WMH volume progression in minor stroke patients could be useful in predicting cognitive decline. Further investigations are needed to explore the risk factors which could be controlled to protect cognitive decline.

## Data Availability

The datasets used and/or analyzed during the current study are available from the corresponding author on reasonable request.
